# Alumist and Neurologist

**Published:** 1881-10-20

**Authors:** 


### *ALUMIST AND NEUROLOGIST.*

The title of an interesting Quarterly edited by C. H. Hughes, M.D., St. Louis, Missouri. The field entered by Dr. Hughes is a complex and difficult one, but we judge from a copy before us that he is fully competent to the task he has assumed. We wish him abundant success.

